# A Systematic Review of the Factors Associated with Post-Traumatic Growth in Parents Following Admission of Their Child to the Intensive Care Unit

**DOI:** 10.1007/s10880-022-09880-x

**Published:** 2022-05-08

**Authors:** S. O’Toole, C. Suarez, P. Adair, A. McAleese, S. Willis, D. McCormack

**Affiliations:** 1 School of Psychology, Queen’s University Belfast, David Keir Building, 18–30 Malone Road, Belfast, BT9 5BN UK; 2 Clinical Psychology Department, Royal Belfast Hospital for Sick Children, Belfast, UK

**Keywords:** Paediatric intensive care, Post-traumatic growth, Parents, Traumatic stress, Critically ill children

## Abstract

This systematic review aims to identify the demographic, clinical and psychological factors associated with post-traumatic growth (PTG) in parents following their child’s admission to the intensive care unit (ICU). Papers published up to September 2021 were identified following a search of electronic databases (PubMed, Medline, Web of Science, PsycINFO, CINAHL, PTSDpubs and EMBASE). Studies were included if they involved a sample of parents whose children were previously admitted to ICU and reported correlational data. 1777 papers were reviewed. Fourteen studies were eligible for inclusion; four were deemed to be of good methodological quality, two were poor, and the remaining eight studies were fair. Factors associated with PTG were identified. Mothers, and parents of older children, experienced greater PTG. Parents who perceived their child’s illness as more severe had greater PTG. Strong associations were uncovered between PTG and post-traumatic stress, psychological well-being and coping. PTG is commonly experienced by this population. Psychological factors are more commonly associated with PTG in comparison with demographic and clinical factors, suggesting that parents’ subjective ICU experience may be greater associated with PTG than the objective reality.

**Supplementary Information:**

The online version contains supplementary material available at 10.1007/s10880-022-09880-x.

## Introduction

The admission of a child to intensive care is a stressful event for families (Bronner et al., 2008; Colville & Gracey, 2006). Admission to the neonatal intensive care unit (NICU) or the paediatric intensive care unit (PICU) is especially distressing for parents and there can be lasting psychological after-effects for them because of this experience (Hill et al., 2018). After-effects of an intensive care admission for parents include anxiety, depression and post-traumatic stress symptoms (Als et al., 2015; Baker & Gledhill, 2017).

Parents experience high rates of trauma exposure whilst in the unit, both via the witnessing of the threat to the life of their child and via exposure to the distress experienced by other children and their families (Colville & Gracey, 2006; Nelson & Gold, 2012). Additionally, parents can find the intensive care environment to be frightening (Dahav & Sjostrom-Strand, 2018; Oxley, 2015). In a recent qualitative investigation, parents described intensive care as “*being in another world*”, with an emphasis on the unit being unpleasant, intense and stressful, and often felt overwhelmed by the technical equipment, noise, medical language and high level of activity in the unit (Dahav & Sjostrom-Strand, 2018, p. 365).

Recent reviews suggest that between 10 and 21% of parents experience persistent post-traumatic stress disorder (PTSD) symptoms following admission of their child to intensive care (Nelson & Gold, 2012), with up to 84% of parents still experiencing these symptoms 3 months following their child’s discharge (Bronner et al., 2008). Factors associated with PTSD symptoms in parents include retrospective reports of stress experienced during admission (Colville & Gracey, 2006), unexpected admission, parents’ degree of worry that their child might die, and the occurrence of another hospital admission/traumatic event after the index admission (Baluffi et al., 2004).

The hallmark symptoms of PTSD, including intrusive re-experiencing, avoidance, and hyperarousal (American Psychiatric Association [APA], 2013), have been well documented amongst parents of NICU children (Aftyka et al., 2014; Lefkowitz et al., 2010) and parents of PICU children (Bronner et al., 2010; Rees et al., 2004). Less precedence has been given to the positive psychological change potentially experienced following a traumatic event, namely, post-traumatic growth (PTG). PTG is a term coined by Tedeschi and Calhoun in 1995, though its concept of origin is timeworn. This phenomenon refers to how an individual may experience positive personal change following life-altering adversity. This positive change can occur across five known domains; (1) having a greater appreciation of life, (2) improved interpersonal relationships, (3) greater perceived personal strength, (4) recognition of new possibilities in one’s life and (5) spiritual or religious growth (Tedeschi & Calhoun, 1996, 2004).

Previous studies have demonstrated that PTG is prevalent in parents of children diagnosed with cancer (Hullmann et al., 2014; Hungerbuehler et al., 2011), type 1 diabetes (Hungerbuehler et al., 2011), and children awaiting corrective surgery for congenital disease (Li et al., 2012), indicating the traumatic nature of such diagnoses. Hungerbuehler et al. (2011) found that parents reported significant levels of PTG 3 years after their child’s diagnosis of diabetes or cancer, but that mothers reported greater levels of PTG than fathers. Levels of PTG were best explained by the quality of family relationships, parents’ psychological distress and children’s medical characteristics (Hungerbuehler et al., 2011). This calls into investigation the acutely traumatic experiences of parents of children with serious paediatric illness and how these may affect levels of PTG in this population.

Significant levels of PTG have previously been reported amongst parents of children with serious paediatric illness (Picoraro et al., 2014); however, a dearth of literature exists examining the development of PTG in parents whose children have been admitted to intensive care specifically. The present systematic review was undertaken to synthesise and critically evaluate the available evidence surrounding factors associated with PTG in parents of children admitted to intensive care. It is important to identify the factors associated with PTG so that we are more aware of parents who are less likely, or more likely, to experience PTG following their NICU or PICU experience with their child (for example, when seeking to employ targeted clinical interventions fostering PTG). This is the first systematic review seeking to corroborate the demographic, clinical and psychological factors associated with parental PTG following such an event.

## Methods

This systematic review was conducted and reported in accordance with the guidelines published on the Preferred Reporting Items for Systematic Reviews and Meta-Analyses (PRISMA) statement (Moher et al., 2009).

### Search Strategy and Study Selection Criteria

A search for potentially eligible papers up to September 2021 was undertaken across seven databases; PubMed, Medline, Web of Science, PsycINFO, CINAHL, PTSDpubs and EMBASE. A combination of controlled vocabulary from databases (e.g. MeSH) and free text words was chosen to reflect the review's focus on the factors associated with PTG in parents whose children have been admitted to NICU or PICU. The search terms used included variations of ‘post-traumatic growth’, ‘parent’, ‘paediatric critical illness’, ‘PICU’ and ‘NICU’. The final search terms used are outlined in Supplementary Table 1.

We included empirical studies that were published in the English language and implemented a quantitative or mixed-methods design, in which (1) PTG was an outcome measure, and (2) demographic (e.g.—age, gender, family type or education level), clinical (e.g.—duration of NICU/PICU stay, gestational age, reason for admission, or severity of illness), or psychosocial data (e.g.—post-traumatic stress, coping, social support, or anxiety) were also reported. We included studies with a population of PICU parents or NICU parents. Studies were excluded if they (1) implemented a qualitative design, (2) featured a different clinical population (e.g.—ICU staff) or (3) reported no PTG-specific data. Reference lists of all eligible research studies and any relevant published reviews were also screened for relevant papers.

**Fig. 1:**
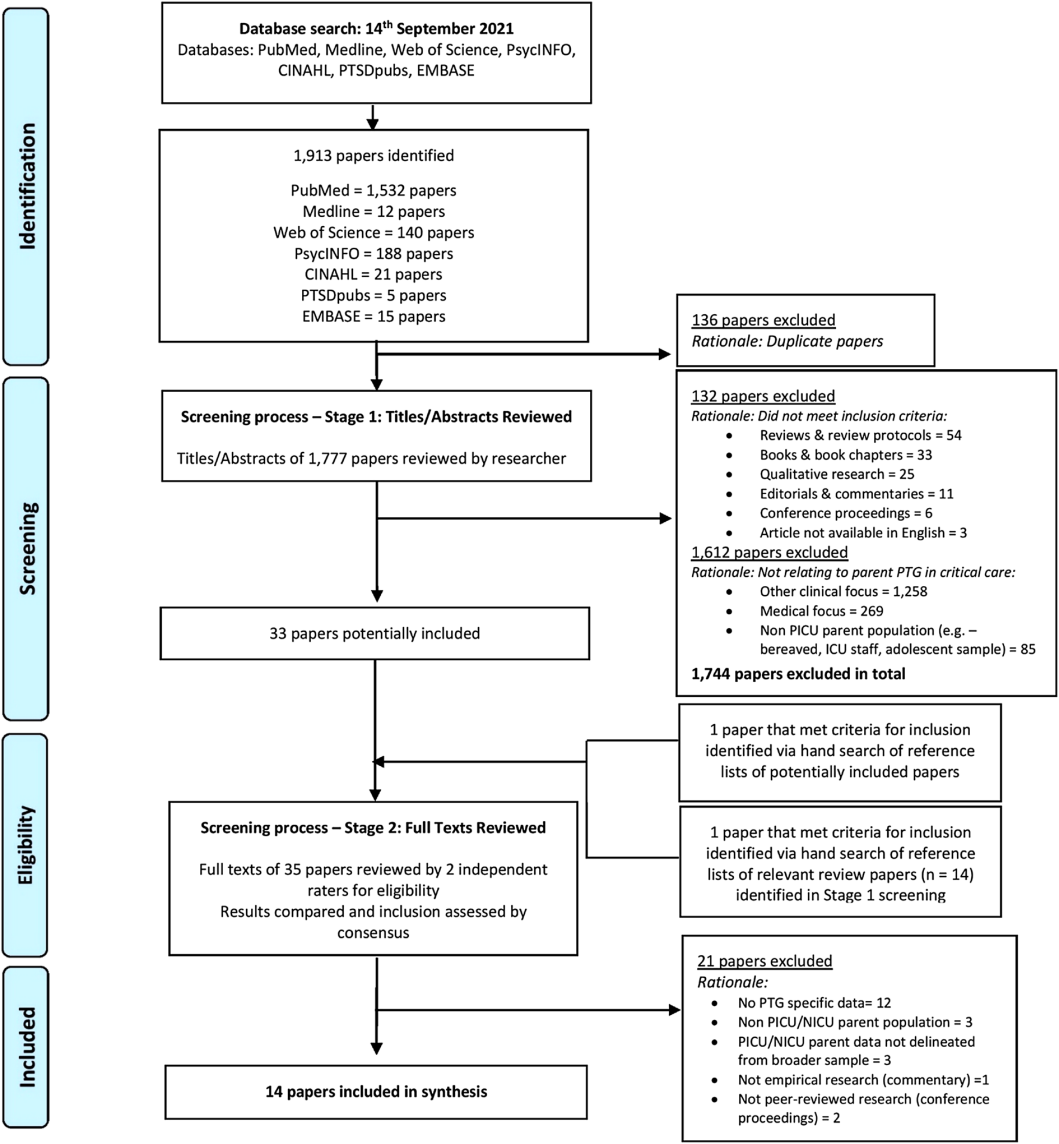
Flow of identification and selection processes (PRISMA diagram)

A PRISMA flow diagram depicting stages of the screening and selection process is presented in Fig. 1. The search strategy yielded 1913 papers for screening. Of 1777 papers retained after duplicates were removed, 1744 were excluded because they did not meet the inclusion criteria. Thirty-three papers were identified as potentially eligible for inclusion. A further two papers were added following (1) manual screening of titles and abstracts of the bibliographies of those potentially included and (2) manual screening of the bibliographies of review papers yielded within the database search. This resulted in screening 35 full-text papers, 21 of which were excluded (see Fig. 1). A total of 14 papers were deemed eligible for inclusion in the review.

Several stages were employed in the screening of papers against the inclusion and exclusion criteria to identify the studies eligible for inclusion in the review. Primarily, the electronic search across the seven databases was completed. Following this, any duplicate papers were identified and removed. The remaining papers underwent a two-stage screening process. In the first stage of screening, the titles and abstracts of all papers were screened by the first author (SOT). In the second stage, the full texts of potentially eligible papers were retrieved and reviewed independently by two review authors (SOT and CS) for eligibility, resulting in 92% interrater agreement (Cohen’s Kappa = 0.83). Two further reviewers (PA and DMcC) resolved any discrepancies through discussion. Reasons for excluding studies at all stages were noted.

### Data Extraction and Synthesis

Results were tabulated to capture the key data extracted from the included studies. The following methodological information was extracted for each study: author, year and country of origin; study aim/objective; study design; data collection method; sample; study setting (NICU or PICU); PTG measure employed; and other variables examined (including demographic, clinical and psychological variables) (see Table 1).

**Table 1:** Characteristics of included studies

Author(s) (year)/*country*	Aim/Objective	Study Design	Data Collection	Setting	Sample	PTG measure(s)	Details of other variable(s)
Aftyka et al. (2020) *Poland*	To determine the predictors of PTG in parents of children hospitalised in the NICU in the past	Cross-sectional quantitative	Survey questionnaires (completed 3 – 12 months following child’s admission to NICU) [*M* = 7 months]	NICU	*n* = 82 parents of infants aged 3–11 months who, in the past, were hospitalised in the NICU 41 mothers and 41 fathers Age of mothers: 14.6% aged 18–24 yrs 85.4% aged 25–39 yrs 0% aged 40–59 yrs Age of fathers: 2.4% aged 18–24 yrs 85.4% aged 25–39 yrs 12.2% aged 40–59 yrs	Post-traumatic Growth Inventory (PTGI) ^a^ [21 items]	Demographic variables Parent age Parent gender Parent education level Number of children Clinical variables Gestational age Birth weight Apgar score 1st minute after birth Duration of NICU stay Survival Infant diagnoses Psychological variables Coping
Aftyka et al. (2017) *Poland*	To determine the incidence and severity of PTG in a group of parents of children hospitalised in the NICU in the past	Cross-sectional quantitative	Survey questionnaires (completed 3 – 12 months following child’s admission to NICU) [*M* = 7 months]	NICU	*n* = 106 parents of infants aged 3–12 months who, in the past, were hospitalised in the NICU 61 mothers and 45 fathers Age: *M* = 31.1, SD = 1.84	Post-traumatic Growth Inventory (PTGI) ^a^ [21 items]	Demographic variables Parent age Parent gender Parent education level Number of children Clinical variables Gestational age Birth weight Apgar score 1st minute after birth Duration of NICU stay Survival Infant diagnoses Psychological variables Posttraumatic Stress Parental Stress Coping
Barr (2011) *Australia*	To explore existential emotion predispositions to guilt, shame, and fear of death and stress-coping strategies as predictors of PTG in parents of infants hospitalized in NICU	Prospective quantitative	Survey questionnaires Time 1: 1 month following onset of hospitalisation Time 2: 13 – 14 months following onset of hospitalisation	NICU	*n* = 158 parents of infants hospitalized for at least 4 days in the NICU 85 mothers and 73 fathers (68 mother–father dyads, 17 individual mothers, 5 individual fathers) Age of mothers: (*M* = 30.8, SD = 5.61) Age of fathers: (*M* = 33.9, SD = 6.40) 69% attained education beyond high school	Post-traumatic Growth Inventory (PTGI) ^a^ [Time 2] [21 items]	Demographic variables Parent age Parent gender Parent ethnicity Parent education level Marital status Clinical variables Duration of NICU stay Severity of the neonatal illness Psychological variables Parental Stress [Time 1] Coping [Time 1] Guilt and shame-proneness [Time 1] Fear of Death [Time 1]
Barr (2015) *Australia*	To explore the relationship between attitudes toward death shortly after leaving the NICU with positive and negative changes in existential outlook one year later	Prospective quantitative	Survey questionnaires -Time 1: 1 to 2 weeks following discharge -Time 2: 1 year following discharge	NICU	*n* = 118 parents of infants who, in the past, were hospitalised for at least 2 days in the NICU -59 mother–father dyads -Age: *M* = 33.1, SD = 5.21 -38.4% (*n* = 43) attained higher education (university degree), 37.5% (*n* = 42) attained technical or higher education diploma -Median length of stay in NICU: 15 days (range: 2 – 70 days)	Positive Changes Scale (CiO-POS) ^b^ (11-item subscale of the Changes in Outlook Questionnaire (CiOQ)) [Time 1 and Time 2]	Demographic variables Parent age Parent gender Parent ethnicity Parent education level Infant gender Clinical variables Gestational age Birth weight APGAR score 1st minute after birth APGAR score 5th minute after birth Duration of NICU stay Infant diagnoses Psychological variables Attitudes towards Death [Time 1]
Barr (2016) *Australia*	To examine the relationship, and moderators of the relationship, between psychological well-being and mental health in parents of sick newborns	Prospective quantitative	Survey questionnaires Time 1: 1 to 2 weeks following discharge Time 2: 1 year following discharge	NICU	*n* = 118 parents of infants who, in the past, were hospitalised for at least 2 days in the NICU 59 mother–father dyads Age: *M* = 33.1, SD = 5.21 38.4% (*n* = 43) attained higher education (university degree), 37.5% (*n* = 42) attained technical or higher education diploma Median length of stay in NICU: 15 days (range: 2 – 70 days)	Positive Changes Scale (CiO-POS) ^b^ (11-item subscale of the Changes in Outlook Questionnaire (CiOQ)) [Time 1 and Time 2]	Demographic variables Parent age Parent gender Parent ethnicity Parent education level Infant gender Clinical variables Gestational age Birth weight APGAR score 1st minute after birth APGAR score 5th minute after birth Duration of NICU stay Infant diagnoses Psychological variables Psychological Well-being [Time 1] Mental Health [Time 2]
Boztepe et al. (2015) *Turkey*	To examine the factors that might predict PTG levels in mothers who had an infant admitted to NICU	Cross-sectional quantitative	Survey questionnaires (completed in the first 12 months following child’s admission to NICU)	NICU	*n* = 210 mothers of infants who were hospitalised in the NICU for at least 1 day in the previous year Age: 12.9% aged 17–22 yrs 39.5% aged 23–28 years 35.7% aged 29–34 years 11.9% aged 35–40 years 45.8% attained education beyond high school 95.7% came from nuclear families 82.9% were receiving support for baby care	Post-traumatic Growth Inventory (PTGI) ^a^ [21 items]	Demographic variables Parent age Parent education level Family type Number of children Number of pregnancies Receiving support for baby care Previous experience of NICU (yes/no) Clinical variables Reason for hospitalisation in NICU Admission time Duration of NICU stay Type of delivery Psychological variables Posttraumatic stress Social support
Brelsford et al., (2020) *USA*	To investigate the relationship between PTG and religiousness and spirituality in parents following their child’s hospitalisation in the NICU	Cross-sectional quantitative	Survey questionnaires (completed 6 weeks following child’s discharge from NICU)	NICU	*n* = 25 parents of infants who, in the past, were hospitalised in the NICU 13 mothers and 12 fathers Age: *M* = 30.36, SD = 4.10	Post-traumatic Growth Inventory (PTGI)^a^ [21 items]	Demographic variables Parent age Parent gender Clinical variables Gestational age Psychological variables Religious coping Spiritual disclosure Theistic sanctification Depression Anxiety Stress
Colville and Cream (2009) *UK*	To establish the degree to which parents report PTG after the intensive care treatment of their child	Prospective quantitative	Survey questionnaires Time 1: At point of discharge from PICU Time 2:4 months following discharge from PICU	PICU	*n* = 50 parents of children admitted to PICU for at least 12 h 40 mothers and 10 fathers (7 mother–father dyads, 33 individual mothers, and 3 individual fathers) Age: Median = 40 years Age range: 23–58 years	-Post-traumatic Growth Inventory (PTGI) ^a^ [21 items] [Time 2]	Demographic variables Parent age Parent gender Child age Child gender Child ethnicity Socioeconomic status (measured using the Townsend Deprivation Index) Clinical variables Duration of PICU stay Emergency admission Ventilation of child llness severity on admission (measured using the Paediatric Index of Mortality [PIM]) Psychological variables Posttraumatic Stress [Time 2] Parental Stress [Time 1] epression [Time 2] Anxiety [Time 2]
Miles et al. (1999) *USA*	To describe the factors predicting maternal adjustment in mothers caring for medically fragile infants	Prospective quantitative	Survey questionnaires, semi-structured interviews, and behavioural observation of dyad interactions: Time 1: Enrolment to study (whilst in NICU) Time 2: At point of discharge from NICU Time 3: 6 months following birth Time 4: 12 months following birth Time 5: 16 months following birth	NICU	*n* = 67 mothers of medically fragile infants requiring hospitalisation Age: *M* = 27 Age range: 16–41 years Education level: *M* = 13 years (range: 8–18 years) 55% were married	A Developmental Impact Rating Scale (developed by the authors for the purpose of this study)^c^ [Time 3, Time 5] [1 item/rating: 7-point scale ranging from negative impact to positive impact (growth)]	Demographic variables Parent age Parent ethnicity Parent education level Marital status Infant age Infant gender Clinical variables Gestational age Birth weight Duration of NICU stay Infant diagnoses Presence of a multi-system diagnosis [Time 1] Level of technology dependence (measured using the Office of Technological Assistance classifications) [Time 1 & 5] Level of mental development (measured using the Mental Development Index [MDI] of the Bayley II Scale) [Time 5] Psychological variables Parental Stress [Time 1] Depression [Time 2 & 4] Caregiver Sense of Control [Time 1 & 4] Satisfaction with Family Life [Time 1 & 4] Worry about Child’s Health [Time 1, 4, & 5] Maternal Identity [Time 1] Maternal Presence [Time 1, 3, & 5] Maternal Competence [Time 1, 3, & 5]
Parker (2016) *USA*	To examine the effects of parental well-being and infant illness severity on changes in parental psychopathology symptoms in the NICU	Prospective quantitative	Survey questionnaires Time 1: Intake interview (whilst in NICU) Time 2: 3 weeks following intake interview	NICU	*n* = 194 parents (97 families) of infants admitted to NICU for at least three weeks 97 mothers and 97 fathers 41% were married	Personal Growth Scale (PGS) ^d^ (4-item subscale of the Psychological Well-being Scales [PWB]) [Time 1, Time 2]	Demographic variables Parent age Parent gender Parent ethnicity Marital status Socioeconomic status (income) Infant age Infant gender Infant ethnicity Clinical variables Duration of NICU stay Surgical intervention Prenatal history Infant diagnoses Severity of infant illness (measured using the Neonatal Therapeutic Intervention Scoring System [NTISS]) Psychological variables Parental Stress [Time 1 & 2] Psychological Well-being [Time 1 & 2] Depression [Time 1 & 2]
Rodríguez-Rey and Alonso-Tapia (2017)^2^ *Spain*	To explore the relationship between PTSD, anxiety, depression, and PTG in parents following their child’s hospitalisation in PICU	Cross-sectional	Survey questionnaires (completed 6 months following child’s discharge from PICU)	PICU	*n* = 143 parents of children who had been admitted to PICU for at least 12 h in the previous 6 months 91 mothers and 52 fathers Age: *M* = 38.24, SD = 6.31	Post-traumatic Growth Inventory (PTGI) ^a^ [21 items]	Demographic variables Parent age Parent gender Child age Child gender CLINICAL variables Illness severity on admission (measured using the Paediatric Index of Mortality II [PIM2]) Parental perception of severity of the child’s condition (Likert rating: 0 – 7) Child diagnosis Duration of PICU stay Reasons for PICU admission Elective admission (yes/no) Psychological variables Posttraumatic Stress Depression Anxiety
Rodríguez-Rey and Alonso-Tapia (2018)^1^ ^2^*Spain*	To explore the degree of parental PTG after a child’s hospitalization in PICU and the role of resilience, emotions, perceived severity of the child’s condition and stress in predicting PTG	Prospective quantitative	Survey questionnaires Time 1: within 48 h following discharge from PICU Time 2: 6 months following discharge from PICU	PICU	*n* = 143 parents of children who had been admitted to PICU for at least 12 h in the previous 6 months 91 mothers and 52 fathers Age: *M* = 38.24, SD = 6.31	-Post-traumatic Growth Inventory (PTGI) ^a^ [21 items] [Time 2]	Demographic variables Parent age Parent gender Child age Child gender Clinical variables Illness severity on admission (measured using the Paediatric Index of Mortality II [PIM2]) Parental perception of severity of the child’s condition (Likert rating: 0 – 7) Child diagnosis Duration of PICU stay Reasons for PICU admission Elective admission (yes/no) Psychological variables Parental Stress [Time 1] Parental Resilience [Time 1] Emotions Experienced [Time 1]
Rozen et al. (2018) *Israel*	To examine the way in which the relationships between the objective severity of premature childbirth, the subjective perception of stress in such circumstances, and several internal and external resources contribute to mothers’ personal growth	Prospective quantitative	Survey questionnaires Time 1: 1 month following birth Time 2: 5 months following birth	NICU	*n* = 94 mothers of preterm infants Age: *M* = 32.54, SD = 3.85 93.1% were married or in a stable relationship 81.9% attained education beyond high school 55.3% first-time mothers	Post-traumatic Growth Inventory (PTGI) ^a^ [Time 2] [21 items]	Demographic variables Parent age Parent education level Parent physical health status Number of children Marital status Socioeconomic status Infant age Infant gender Clinical variables Gestational age Birth weight Infants’ medically defined risk level (moderate-high risk *or* low risk) Type of delivery Ventilation Psychological variables Parental stress [Time 1] Self-esteem [Time 1] Attachment style [Time 1] Perceived emotional support [Time 1]
Taubman-Ben-Ari et al., (2010) *Israel*	To examine factors (such as internal resources, external resources, and features of the birth itself) that might contribute to a mother’s personal growth after the birth of preterm twins	Prospective quantitative	Survey questionnaires Time 1: 3 weeks following birth Time 2: 1 year following birth	NICU	*n* = 64 mothers of preterm twins Age: *M* = 30.31, SD = 4.20) 100% were married or cohabitating with a male partner	Post-traumatic Growth Inventory (PTGI) ^a^ [Time 2] [21 items]	Demographic variables Parent age Parent education level Parent occupation Marital status Socioeconomic status Infant age Infant gender Clinical variables None reported Psychological variables Parental stress [Time 1] Attachment style [Time 1] Psychological well-being [Time 1] Social support (Maternal Grandmother) [Time 1] Marital adaptation [Time 1] Infant temperament [Time 1] Feelings/Concerns towards baby [Time 1]

^a^Post-traumatic Growth Inventory (PTGI), developed byTedeschi and Calhoun (1996)

^b^Positive Changes Scale (CiO-POS) (11-item subscale of the Changes in Outlook Questionnaire (CiOQ)), developed by Joseph et al. (1993)

^c^Developmental Impact Rating Scale [developed by the authors (Miles et al., 1999) for the purpose of the included study]

^d^Personal Growth Scale (PGS) (4-item subscale of the Psychological Well-being Scales [PWB]), developed by Ryff et al. (2006) [adapted from Ryff (1989)]

The following key findings were extracted for each study: levels of PTG, demographic factors associated with PTG, clinical factors associated with PTG and psychological factors associated with PTG (see Table 2). All data were extracted independently by two reviewers (SOT and CS) and cross-checked by two other reviewers (PA and DMcC) for accuracy with any discrepancies resolved through discussion. Because of methodological heterogeneity uncovered within the included studies, and variability in the method of reporting psychometric outcomes, a narrative analysis was conducted across all findings.

**Table 2:** Key findings pertaining to factors associated with PTG

Author(s) (year)	Levels of PTG *M* (SD)	Demographic factors associated with PTG	Clinical factors associated with PTG	Psychological factors associated with PTG
Aftyka et al. (2020)	Total PTG ^a^: Mothers: *2.89* (1.32) Fathers: *3.05* (0.88) Four Factors Structure: - Changes in self-perception: *3.56* (1.42) in mothers *2.85* (0.88) in fathers - Relating to others: *2.89* (1.12) in mothers *3.27* (0.96) in fathers - Appreciation of life: *3.44* (1.08) in mothers *3.56* (1.05) in fathers - Spiritual change: *3.85* (1.13) in mothers *2.43* (1.23) in fathers	None reported	None reported	Coping PTG *correlated significantly and positively* with: Active coping in mothers (*r* = .50, *p* < .01)** Planning in mothers (*r* = .44, *p* < .01)** Use of instrumental social support in mothers (*r* = .51, *p* < .01)** Use of emotional social support in mothers (*r* = .49, *p* < .01)** Use of emotional social support in fathers (*r* = .36, *p* < .05)* Suppression of competing activities in mothers (*r* = .41, *p* < .01)** Positive reinterpretation and growth in mothers (*r* = .35, *p* < .05)* Positive reinterpretation and growth in fathers (*r* = .34, *p* < .05)* Focus on and venting of emotions in mothers (*r* = .39, *p* < .05)*
Aftyka et al. (2017)	**Total PTG **^**a**^**:** *66.66* (19.77) Four Factors Structure: Personal strength: *2.93* (1.10) Relating to others: *3.38* (1.04) Appreciation of life: *3.77* (1.09) Spiritual change: (1.44)	None reported	Infant survival Parents of children who survived had *significantly higher* PTG (*M* = 68.63, SD = 18.89) than the parents of children who died (*M* = 42.50, SD = 14.00) (*p* < .001)***	Posttraumatic stress PTG *correlated significantly and positively* with: Posttraumatic stress symptoms (*r* = .22, *p* < .05)* Intrusion (*r* = .26, *p* < .01)** Coping PTG *correlated significantly and positively* with: Positive reinterpretation and growth strategy (*r* = .34, *p* < .001)*** Task-oriented coping (*r* = .29, *p* < .01)** Avoidance-oriented coping (*r* = .25, *p* < .01)**
Barr (2011)	Total PTG ^a^: Mothers: *55.0* (24.05) Fathers: *47.7* (23.34)	None reported	None reported	Coping PTG *correlated significantly and positively* with: Confrontive coping in mothers (*r* = .32, *p* < .01)** Confrontive coping in fathers (*r* = .26, *p* < .05)* Self-controlling in mothers (*r* = .38, *p* < .001)*** Self-controlling in fathers (*r* = .24, *p* < .05)* Seeking social support in mothers (*r* = .33, *p* < .01)** Accepting responsibility in mothers (*r* = .43, *p* < .001)*** Escape avoidance in mothers (*r* = .36, *p* < .01)** Planful problem solving in mothers (*r* = .30, *p* < .01)** Positive reappraisal in mothers (*r* = .56, *p* < .001)*** Positive reappraisal in fathers (*r* = .44, *p* < .001)*** Parental stress PTG *correlated significantly and positively* with: Parental stress in mothers (relating to the NICU environment) (*r* = .32, *p* < .01)** Parental stress in fathers (relating to the NICU environment) (*r* = .26, *p* < .05)* Guilt and shame-proneness PTG *correlated significantly and positively* with: Guilt-proneness in fathers (*r* = .25, *p* < .05)* Fear of death PTG *correlated significantly and positively* with: Fear of death in fathers (*r* = .37, *p* < .01)**
Barr (2015)^1^	Total CiO-POS^b^: Mothers: *49.3* (8.40) Fathers: *45.9* (9.74)	Parent gender Mothers were *significantly more likely* to experience positive changes in outlook (*M* = 49.3, SD = 8.40) than fathers (*M* = 45.9, SD = 9.74) [*t* (117) = 2.82, *p* < .01] **	None reported	Attitudes towards death Positive changes in outlook (CiO-POS) *correlated significantly and positively* with Death avoidance in mothers (*r* = .31, *p* < .05)* Death avoidance in fathers (*r* = .44, *p* = < .001)*** Positive changes in outlook (CiO-POS) *correlated significantly and negatively* with Escape acceptance in mothers (*r* = -.26, *p* < .05)*
Barr (2016)^2^	Total CiO-POS^b^: Mothers: *49.0* (7.32) Fathers: *47.0* (9.98)	None reported	None reported	Psychological wellbeing Positive changes in outlook (CiO-POS) *correlated significantly and positively* with Psychological well-being in mothers (*r* = .44, *p* < .01)** Mental health Positive changes in outlook (CiO-POS) *correlated significantly and positively* with Positive mental health in mothers (*r* = .37, *p* < .01)**
Boztepe et al (2015)	**Total PTG**^**a**^**: ***75.70* (22.17)	None reported	None reported	Posttraumatic Stress The following variables *significantly predicted* PTG in mothers: Impact of event relief (*p* < .01)** Impact of event escape (*p* < .001)*** Impact of event over-stimulation (*p* < .01)** Social support The following variables *significantly predicted* PTG in mothers: Social support from family (*p* < .05)* Social support from friends (*p* < .05)* Social support from a significant other (*p* < .05)*
Brelsford et al. (2020)	**Total PTG **^**a**^**:** *52.16* (27.37)	None reported	None reported	Religious coping PTG *correlated significantly and positively* with: Positive religious coping (*r* = .41, *p* < .05)* Spiritual disclosure PTG *correlated significantly and positively* with: Spiritual disclosure (*r* = .43, *p* < .05)* Theistic sanctification PTG *correlated significantly and positively* with: Theistic sanctification (*r* = .52, *p* < .05)* Non-theistic sanctification (*r* = .73, *p* < .001)*** Stress PTG *correlated significantly and positively* with: Stress (*r* = .46, *p* < .05)*
Colville and Cream (2009)	**Total PTG**^**a**^**:** *49.0* (23.9)	Child age Parents of older children reported *significantly higher* PTG than those whose children were younger (*p* < .05)*	Ventilation Parents of children who were ventilated reported significantly higher PTG than those who weren’t (*p* < .05)*	Posttraumatic stress PTG *correlated significantly and positively* with: Posttraumatic stress symptoms 4 months following discharge from PICU (*p* < .05)*
Miles et al. (1999)	PTG^c^ at Time 3: *4.5* (1.3) *(indicating an outcome between neutral and slightly negative)* PTG^c^ at Time 5: *5.3* (1.3) (*indicating an outcome between neutral and slightly positive*)	None reported	Mental development Mothers of children with lower mental development were *significantly less likely* to experience positive growth (*p* < .01)** Level of technology dependence Mothers of children who were more technology-dependent at an early age were *significantly less likely* to experience positive growth (*p* < .05)*	Maternal identity Mothers of children with higher parental identity in the early months of life were *significantly less likely* to experience positive growth (*p* < .05)* Worry about child’s health Mothers of children with more worry about their child’s health were *significantly less likely* to experience positive growth (*p* < .001)***
Parker (2016)	Total PG^d^: *4.82* (0.59)	None reported	None reported	Psychological wellbeing Personal growth *correlated significantly and positively* with: Autonomy (*r* = .45, *p* < .05)* Environmental mastery (*r* = .58, *p* < .05)* Positive relations with others (*r* = .47, *p* < .01)** Purpose in life (*r* = .63, *p* < .05)* Self-acceptance (*r* = .55, *p* < .05)* Parental stress Personal growth [Time 1] was *significantly and negatively* correlated with symptoms of acute stress disorder (at Time 2) (*r* = −.50, *p* < .01)**
Rodríguez-Rey and Alonso-Tapia (2017)^3^	Total PTG^a^: *47.40* (26.74) Five-factor structure 54.5% of parents endorsed* ***appreciation for life*** [*8.75* (4.46) in mothers, *7.25* (4.53) in fathers] 46.2% of parents endorsed ***personal strength*** [*10.50* (5.48) in mothers *7.75* (6.18) in fathers] 40.6% of parents endorsed ***relating to others*** [*14.31* (10.16) in mothers *17.38* (9.58) in fathers) 29.4% of parents endorsed ***new possibilities*** [*9.59* (6.86) in mothers *8.02* (6.59) in fathers] 25.9% of parents endorsed ***spiritual change*** [*2.25* (2.97) in mothers, *3.15* (3.38) in fathers] Three-factor structure used: 44.8% of parents endorsed ***personal growth*** 54.5% of parents endorsed ***interpersonal growth*** 21% of parents endorsed ***transpersonal growth***	None reported	Parental perception of illness severity Parents of children who perceived their illness as more severe were *significantly more likely* to experience interpersonal growth (*r* = .19, *p* ≤ .05)*	Posttraumatic stress PTG (overall) *correlated significantly and positively* with: Posttraumatic stress symptoms (*r* = .28, *p* ≤ .001)*** Intrusion (*r* = .27, *p* ≤ .001)*** Avoidance (*r* = .22, *p* ≤ .01)** Hyper-activation (*r* = .27, *p* ≤ .001)*** Interpersonal growth *correlated significantly and positively* with: Posttraumatic stress symptoms (*r* = .32, *p* ≤ .001)*** Intrusion (*r* = .32, *p* ≤ .001)*** Avoidance (*r* = .27, *p* ≤ .001)*** Hyper-activation (*r* = .30, *p* ≤ .001)*** Transpersonal growth *correlated significantly and positively* with: Posttraumatic stress symptoms (*r* = .31, *p* ≤ .001)*** Intrusion (*r* = .29, *p* ≤ .001)*** Avoidance (*r* = .18, *p* ≤ .05)* Hyper-activation (*r* = .29, *p* ≤ .001)*** Anxiety PTG (overall) *correlated significantly and positively* with symptoms of anxiety (*r* = .22, *p* ≤ .01)** Interpersonal growth *correlated significantly and positively* with symptoms of anxiety (*r* = .22, *p* ≤ .01)* Transpersonal growth *correlated significantly and positively* with symptoms of anxiety (*r* = .29, *p* ≤ .001)*** Depression PTG (overall) *correlated significantly and positively* with symptoms of depression (*r* = .20, *p* ≤ .05)* Interpersonal growth *correlated significantly and positively* with symptoms of depression (*r* = .17, *p* ≤ .05)* Transpersonal growth *correlated significantly and positively* with symptoms of depression (*r* = .31, *p* ≤ .001)***
Rodríguez-Rey and Alonso-Tapia (2018)^2^	Total PTG^a^: *47.40* (26.74) Five-factor structure: 54.5% of parents endorsed* ***appreciation for life*** [*8.75* (4.46) in mothers, *7.25* (4.53) in fathers] 46.2% of parents endorsed ***personal strength*** [*10.50* (5.48) in mothers *7.75* (6.18) in fathers] 40.6% of parents endorsed ***relating to others*** [*14.31* (10.16) in mothers *17.38* (9.58) in fathers) 29.4% of parents endorsed ***new possibilities*** [*9.59* (6.86) in mothers *8.02* (6.59) in fathers] 25.9% of parents endorsed ***spiritual change*** [*2.25* (2.97) in mothers, *3.15* (3.38) in fathers] Three-factor structure: 44.8% of parents endorsed ***personal growth*** 54.5% of parents endorsed ***interpersonal growth*** 21% of parents endorsed ***transpersonal growth***	Parent gender Mothers reported *significantly higher* personal strength (*M* = 10.59, SD = 5.48) than fathers (*M* = 7.75, SD = 4.53) (*p* ≤ .05)*	Parental perception of illness severity Parents of children who perceived their child’s illness as more severe were *significantly more likely* to experience PTG (*r* = .21, *p* ≤ .05)*	Parental stress PTG *correlated significantly and positively* with: Parental stress relating to the PICU environment (*r* = .22, *p* ≤ .01)** Emotions experienced PTG *correlated significantly and positively* with: Positive emotions experienced during admission (*r* = .20, *p* ≤ .05)*
Rozen et al. (2018)	Total PTG^a^: Not reported For the purpose of this review, calculated from five domains as: *2.97* (1.39) Five PTG domains: Relations with others *2.99* (0.95) New possibilities *2.83* (1.11) Personal strength *3.5* (1.13) Spirituality *1.99* (1.76) Appreciation of life *3.46* (1.29)	Education level Personal strength correlated *significantly and negatively* with parent education level (*t* = − 2.81, *p* ≤ .01)** Spirituality correlated *significantly and negatively* with parent education level (*t* = − 2.28, *p* ≤ .05)* Socioeconomic status Relations with others correlated *significantly and negatively* with parent socioeconomic status (*t* = − 2.49, *p* ≤ .05)*	Infants’ medically defined risk level Mothers of infants at moderate-high risk were *significantly more likely* to experience greater spirituality than mothers of low-risk infants (*F* = 8.26, *p* ≤ .01)**	**Attachment Style** Personal strength correlated *significantly and negatively* with mothers’ attachment anxiety (*r* = − .22, *p* < .05)* Spirituality correlated *significantly and negatively* with mothers’ attachment anxiety (*r* = − .23, *p* < .05)* Perceived emotional support Relations with others correlated *significantly and positively* with maternal emotional support (*r* = .32, *p* < .01)** New possibilities correlated *significantly and positively* with maternal emotional support (*r* = .27, *p* < .05)* Personal strength correlated *significantly and positively* with maternal emotional support (*r* = .44, *p* < .01)** Spirituality correlated *significantly and positively* with maternal emotional support (*r* = .23, *p* < .05)* Appreciation of life correlated *significantly and positively* with maternal emotional support (*r* = .25, *p* < .05)*
Taubman-Ben-Ari et al., (2010)	Total PTG^a^: *3.27* (0.71)	None reported	None reported	Marital adaptation PTG was significantly and positively correlated with better marital adaptation (in mothers) immediately following birth (*r* = .30, *p* < .05)*

*p < .05, **p < .01, ***p < .001

^a^Post-traumatic Growth Inventory (PTGI), developed by Tedeschi and Calhoun (1996)

^b^Positive Changes Scale (CiO-POS) (11-item subscale of the Changes in Outlook Questionnaire (CiOQ)), developed by Joseph et al. (1993)

^c^Developmental Impact Rating Scale [developed by the authors (Miles et al., 1999) for the purpose of the included study]

^d^Personal Growth Scale (PGS) (4-item subscale of the Psychological Well-being Scales [PWB]), developed by Ryff et al. (2006) [adapted from Ryff (1989)]

^1^The same sample of participants was examined in the following papers, as confirmed in correspondence with the author; Barr (2015) and Barr (2016)

^2^The same sample of participants was examined in the following papers, as confirmed in correspondence with the author(s); Rodríguez-Rey and Alonso-Tapia (2017) and Rodríguez-Rey and Alonso-Tapia (2018)

### Quality Assessment

The quality of included studies was assessed independently by two review authors (SOT and CS), with two other reviewers (PA and DMcC) resolving any disagreements by discussion and consensus. As all the studies included in the present review employed quantitative methods, the Quality Assessment Tool for Observational and Cross-Sectional Studies, developed by the National Heart, Lung and Blood Institute [NHLBI], was used to assess study quality and risk of bias (NHLBI & accessed, 2019). This tool aims to address common issues arising in cross-sectional and observational studies. The tool contains 14 questions, for which each rater can assign the answers “yes”, “no”, “CD” (cannot determine), “NA” (not applicable) or “NR” (not reported). The tool enabled the assignment of a rating of ‘poor’, ‘fair’ or ‘good’ to each study.

## Findings

### Description of the Included Studies

Characteristics of the included studies are outlined in Table 1. The 14 papers included in this review were published between 1999 and 2020, with twelve of the studies published within the last 10 years. The studies involved a total of 1311 participants and all employed either a cross-sectional or prospective quantitative study design. The studies were conducted in Australia, the United States, Spain, Israel, the United Kingdom, Poland and Turkey.

Considering the samples employed in the studies, the number of mothers (*n* = 922) far outweighed fathers (*n* = 389). This is because four of the included studies examined a mother-only sample (Boztepe, et al., 2015; Miles et al., 1999; Rozen et al., 2018; Taubman-Ben-Ari et al., 2010). Six studies looked at mother and father data combined (Aftyka et al., 2017; Brelsford et al., 2020; Colville & Cream, 2009; Parker, 2016; Rodriguez-Rey & Alonso-Tapia, 2017, 2018), and four studies examined data from mothers and fathers separately (Aftyka et al., 2020; Barr, 2011, 2015, 2016). Overall, participant numbers in studies ranged from 25 to 210. A greater number of studies investigated PTG in a population of NICU parents (Aftyka et al., 2017, 2020; Barr, 2011, 2015, 2016; Boztepe, et al., 2015; Brelsford et al., 2020; Miles et al., 1999; Parker, 2016; Rozen et al., 2018; Taubman-Ben-Ari et al., 2010), compared to a population of PICU parents (Colville & Cream, 2009; Rodriguez-Rey & Alonso-Tapia, 2017, 2018).

Twelve of the included studies identified the PTG in parents whose child had been admitted to intensive care as a primary focus. The aims and objectives of the studies varied greatly, with much heterogeneity amongst the variables of interest. For example, nine studies broadly investigated how different psychological and clinical factors are associated with PTG and parental adjustment following intensive care, two studies evaluated the incidence and severity of PTG amongst parents following intensive care, one study examined the risk factors associated with PTSD and PTG in these parents and finally, two studies explored psychological well-being of this population in general. The psychometric tools used to measure PTG in the studies included the following: The Post-traumatic Growth Inventory [PTGI] (Aftyka et al., 2017, 2020; Barr, 2011; Boztepe, et al., 2015; Brelsford et al., 2020; Colville & Cream, 2009; Rodriguez-Rey & Alonso-Tapia, 2017, 2018; Rozen et al., 2018; Taubman-Ben-Ari et al., 2010), the Positive Changes subscale of the Changes in Outlook Questionnaire [CiO-POS] (Barr, 2015, 2016), the Personal Growth subscale of the Psychological Well-being Scales [PGS] (Parker, 2016) and a Developmental Impact Rating Scale developed by the authors for the purpose of measuring growth (Miles et al., 1999).

### Methodological Quality of the Included Studies

Two reviewers (SOT and CS) independently rated the quality of each study resulting in 92% interrater agreement (Cohen’s Kappa of 0.87). Use of the Quality Assessment Tool for Observational and Cross-Sectional Studies deemed eight studies to be of “fair” quality and four studies to be of “good” quality. Two studies were assigned the rating “poor” (See Table 3).

**Table 3:** Methodological quality assessment of included studies

	Research question	Study population	Participation rate	Recruitment	Sample size justification	Exposure assessed	Sufficient timeframe	Levels of exposure	Exposure measures	Repeated assessment	Outcome measures	Blinding	Follow-up rate	Statistical analyses	Rating
Aftyka et al. (2020)	Yes	Yes	Yes	NR	No	No	No	No	Yes	NA	Yes	NA	NA	No	Fair
Aftyka et al. (2017)	Yes	Yes	No	NR	No	No	No	No	Yes	NA	Yes	NA	NA	No	Poor
Barr (2011)	Yes	Yes	Yes	NR	No	Yes	No	No	Yes	No	Yes	NA	NR	Yes	Fair
Barr (2015)	Yes	No	Yes	NR	No	No	No	No	Yes	No	Yes	NA	Yes	Yes	Fair
Barr (2016)	Yes	Yes	Yes	Yes	No	No	No	No	Yes	No	Yes	NA	Yes	Yes	Good
Boztepe et al. (2015)	Yes	Yes	No	NR	Yes	No	No	No	Yes	NA	Yes	NA	NA	No	Fair
Brelsford et al. (2020)	Yes	Yes	No	Yes	No	No	No	No	Yes	A	es	A	NA	No	Fair
Colville and Cream (2009)	Yes	Yes	Yes	Yes	No	Yes	No	Yes	Yes	Yes	Yes	NA	No	No	Good
Miles et al. (1999)	Yes	No	NR	NR	No	Yes	No	No	Yes	No	No	NA	Yes	No	Poor
Parker (2016)	Yes	No	NR	NR	Yes	Yes	No	No	Yes	Yes	Yes	NA	NR	Yes	Fair
Rodríguez-Rey and Alonso-Tapia (2017)	Yes	No	Yes	Yes	No	No	No	No	Yes	NA	Yes	NA	NA	No	Fair
Rodríguez-Rey and Alonso-Tapia (2018)	Yes	Yes	Yes	Yes	NR	Yes	No	Yes	Yes	No	Yes	NA	No	No	Good
Rozen et al. (2018)	Yes	Yes	Yes	Yes	Yes	Yes	No	Yes	Yes	No	Yes	NA	Yes	Yes	Good
Taubman-Ben-Ari et al. (2010)	Yes	No	Yes	NR	No	Yes	No	No	No	No	Yes	NA	Yes	Yes	Fair

**Research question:** Was the research question or objective in this paper clearly stated?

**Study population:** Was the study population clearly specified and defined?

**Participation rate**: Was the participation rate of eligible persons at least 50%?

**Recruitment:** Were all the subjects selected or recruited from the same or similar populations (including the same time period)? Were inclusion and exclusion criteria for being in the study pre-specified and applied uniformly to all participants?

**Sample size justification:** Was a sample size justification, power description, or variance and effect estimates provided?

**Exposure assessed prior:** For the analyses in this paper, were the exposure(s) of interest measured prior to the outcome(s) being measured?

**Sufficient timeframe:** Was the timeframe sufficient so that one could reasonably expect to see an association between exposure and outcome if it existed?

**Levels of exposure:** For exposures that can vary in amount or level, did the study examine different levels of the exposure as related to the outcome (e.g. categories of exposure, or exposure measured as continuous variable)?

**Exposure measures:** Were the exposure measures (independent variables) clearly defined, valid, reliable, and implemented consistently across all study participants?

**Repeated assessment:** Was the exposure(s) assessed more than once over time?

**Outcome measures:** Were the outcome measures (dependent variables) clearly defined, valid, reliable, and implemented consistently across all study participants?

**Blinding:** Were the outcome assessors blinded to the exposure status of participants?

**Follow-up rate:** Was loss to follow-up after baseline 20% or less?

**Statistical analyses:** Were key potential confounding variables measured and adjusted statistically for their impact on the relationship between exposure(s) and outcome(s)?

Common issues arising amongst studies included the absence of pre-specified inclusion and exclusion criteria for recruitment (*n* = 8) and a lack of reporting sample size justification and power calculations (*n* = 11). Additionally, no studies incorporated a sufficient timeframe for observing the development of PTG within a population of parents whose child was admitted, or had previously been admitted, to intensive care (*n* = 14). The longest timeframe employed within the included studies was a follow-up of 16 months following the child’s birth (with baseline measurement occurring at time of birth) (Miles et al., 1999). The median timeframe employed in the studies was 7.5 months.

### Levels of PTG Amongst Parents Following Admission of their Child to the Intensive Care Unit

Parent samples in all the included studies endorsed a medium to very high level of PTG in the weeks, months and years following their child’s admission to intensive care. Due to variability in both the psychometric tools used, and the method of reporting, providing an estimate of the average prevalence of PTG within all study samples presented a challenge. Studies employing the PTGI measure that reported a mean total PTG demonstrated scores that ranged from 47.4 to 75.70, indicating a moderate to very high degree of PTG (Aftyka et al., 2017, 2020; Barr, 2011; Boztepe et al., 2015; Brelsford et al., 2020; Colville & Cream, 2009; Rodríguez-Rey & Alonso-Tapia, 2017, 2018; Rozen et al., 2018; Taubman-Ben-Ari et al., 2010).

### What are the Demographic Factors Associated with PTG in Parents Following Admission of Their Child to the Intensive Care Unit?

Four studies demonstrated significant associations between PTG and demographic factors, including parent gender (Barr, 2015; Rodriguez-Rey & Alonso-Tapia, 2018), child age (Colville & Cream, 2009), parent education level (Rozen et al., 2018) and socioeconomic status (Rozen et al., 2018). Mothers of children previously admitted to intensive care were significantly more likely to experience higher PTG than fathers [*p* < .01; *p* ≤ .05] (Barr, 2015; Rodriguez-Rey & Alonso-Tapia, 2018). Furthermore, parents of older children exhibited significantly higher levels of PTG following their child’s PICU admission, when compared to parents of younger children [*p* < .05] (Colville & Cream, 2009). Finally, in their 2018 study, Rozen et al. highlighted associations between PTG and parent education level; with parents with lower education levels endorsing greater personal growth [*p* ≤ .01] and spirituality [*p* ≤ .05] (Rozen et al., 2018). Parents of lower socioeconomic status also reported greater growth in interpersonal relationships [*p* ≤ .05] (Rozen et al., 2018).

### What are the Clinical Factors Associated with PTG in Parents Following Admission of Their Child to the Intensive Care Unit?

Six studies demonstrated significant associations between PTG and clinical factors including parental perceptions of child’s illness severity (Rodriguez-Rey & Alonso-Tapia, 2017, 2018), child’s illness severity (Rozen et al., 2018), child ventilation (Colville & Cream, 2009), child level of technology dependence (Miles et al., 1999), child’s mental development (Miles et al., 1999) and child survival (Aftyka et al., 2017). Parents who perceived their child’s illness to be more severe [*p* ≤ .05], and parents whose children were objectively more severely-ill [*p* ≤ .01], were demonstrated as significantly more likely to experience PTG (Rodriguez-Rey & Alonso-Tapia, 2017, 2018; Rozen et al., 2018). Colville and Cream (2009) found that parents of ventilated children exhibited higher PTG scores than parents of non-ventilated children [(*p* < .05]. However, in another study, mothers of children who were more technology-dependent (i.e.—had a greater number of technologies involved in their care) at an early age were significantly less likely to experience PTG when compared to mothers whose children were less dependent [*p* < .05] (Miles et al., 1999). Mothers of children with lower mental development were also significantly less likely to experience PTG [*p* < .01] (Miles et al., 1999). Finally, Aftyka et al. (2017) found that parents of children who survived following their admission to intensive care experienced greater levels of PTG than bereaved parents [*p* < .001].

### What are the Psychological Factors Associated with PTG in Parents Following Admission of Their Child to the Intensive Care Unit?

All studies included in this review demonstrated significant associations between PTG and psychological factors. In total, thirty-nine psychometric tools were used to examine a wide array of psychological variables. A full breakdown of the psychometric tools used to examine each variable is outlined in Supplementary Table 2.

#### Demonstrated Associations Between PTG and Post-Traumatic Stress

The psychological presentation with the greatest links to PTG amongst parents was post-traumatic stress. Symptoms of post-traumatic stress, including intrusions, avoidance and hyperarousal, were found to be significantly positively associated with PTG in all the studies in which they were examined (Aftyka et al., 2017; Boztepe et al., 2015; Colville & Cream, 2009; Rodriguez-Rey & Alonso-Tapia, 2017), with particularly strong links to interpersonal and transpersonal growth uncovered (Rodriguez-Rey & Alonso-Tapia, 2017). Consistent with the available literature, Colville and Cream (2009) found the relationship between PTG and PTSD symptoms to be curvilinear; suggesting that parents experiencing a moderate level of PTSD are more likely to exhibit PTG when compared to those experiencing lower or higher levels of post-traumatic stress. However, this curvilinear relationship was contraindicated in Rodriguez-Rey and Alonso-Tapia’s (2017), the results of which show that high levels of psychopathology are also linked to PTG. Given that PTG is more strongly linked to positive outcomes over 2 years following the traumatic event (Helgeson et al., 2006), and these studies measure PTG at 4 months and 6 months post-admission, respectively, the nature of the relationship between PTG and PTSD warrants further longitudinal investigation.

#### Demonstrated Associations Between PTG and Parental Stress

Environmental stressors in both the NICU environment (e.g. the sights and sounds of the unit and infants’ appearance) (Barr, 2011), and the PICU environment (e.g. medical procedures conducted on the child and separation from the child) (Rodriguez-Rey & Alonso-Tapia, 2018), were found to be predictors of PTG in parents of admitted children, particularly mothers (Barr, 2011). Rodriguez-Rey and Alonso-Tapia (2018) also found that positive emotions experienced during admission were positively related to PTG 6 months following discharge [*r* = .20, *p* ≤ .05]. Conversely, parents who experienced greater worry in relation to their child’s health were less likely to experience PTG during the first 16 months following their child’s admission [*p* < .001] (Miles et al., 1999).

Parker (2016) also demonstrated that personal growth at time of admission to intensive care was significantly negatively correlated with the symptoms of acute stress disorder at 3-week follow-up [*r* = − .50, *p* < .01]. Whilst not explored in other studies, this finding indicates that personal growth (being open to new experiences and considering the self as growing and expanding over time) may actually be a protective factor against the development of acute stress disorder amongst these parents.

#### Demonstrated associations Between PTG and Parent Psychological Well-Being

Greater psychosocial well-being and more positive mental health in mothers were significantly (moderately) associated with positive changes in outlook in mothers [*r* = .44, *p* < .01], but not in fathers (Barr, 2016). Parker (2016) replicated this finding across four domains of psychological well-being [all *p* < .05], identifying a particularly strong association between positive relations with others and personal growth [*r* = .47, *p* < .01].

Considering psychological presentations and how these are linked to PTG, Rodriguez-Rey and Alonso-Tapia (2017) found PTG to be significantly and positively correlated with both anxiety [*r* = .22, *p* ≤ .01] and depression [*r* = .20, *p* ≤ .05]. Interestingly, transpersonal growth (encompassing spiritual beliefs and life possibilities) was most strongly associated with greater anxiety [*r* = .29, *p* ≤ .001] and depression [*r* = .31, *p* ≤ .001] in parents of children whose children had been admitted to intensive care (Rodriguez-Rey & Alonso-Tapia, 2017). These findings add weight to the evidence that positive and negative effects of traumatic events (such as a child’s intensive care hospitalisation) can coexist and manifest within the same person.

#### Demonstrated Associations Between PTG and Coping Strategies

Several significant positive relationships were uncovered between PTG and the use of adaptive coping strategies. The most strongly associated coping strategies were those of positive reinterpretation (Aftyka et al., 2017), planful problem-solving and positive reappraisal (Barr, 2011) [all *p* < .001], with moderate associations revealed between PTG and task-oriented coping, and PTG and avoidance-oriented coping [both *p* < .01] (Aftyka et al., 2017).

Coping strategies were more commonly linked to greater PTG in mothers rather than fathers (Aftyka et al., 2020; Barr, 2011). Gender differences were found in the type of coping strategies adopted by mothers (accepting responsibility [*r* = .43, *p* < .001], planful problem-solving [*r* = .30, *p* < .01], escape avoidance [*r* = .36, *p* < .01], social support seeking [*r* = .33, *p* < .01], confrontive coping [*r* = .32, *p* < .01], active coping [*r* = .50, *p* < .01], planning [*r* = .44, *p* < .01], suppression of competing activities [*r* = .41, *p* < .01] and focus on and venting of emotions [*r* = v39, *p* < .05]) versus fathers (confrontive coping [*r* = .26, *p* < .05]), with self-controlling linked to greater PTG in both mothers [*r* = .38, *p* < .001] and fathers [*r* = .24, *p* < .05] (Aftyka et al., 2020; Barr, 2011).

#### Demonstrated Associations Between PTG and Social Support

One study identified a positive relationship between PTG and social support, indicating that parents who perceived more social support from their friends, family and significant other were more likely to experience PTG [all *p* < .05] (Boztepe et al., 2015).

#### Demonstrated Associations Between PTG and Attitudes Towards Death

Barr (2011, 2015) sought to investigate parents’ attitudes towards death (including how much they both feared and accepted death) and how these may be related to PTG following their child’s admission to intensive care. Gender differences in attitudes towards death were revealed; escape acceptance (i.e.—accepting death as the ultimate solution to life’s problems and worries) was significantly negatively correlated with PTG in mothers [*r* = − .26, *p* < .05] (Barr, 2015), whereas fearing death was significantly positively associated with PTG in fathers [*r* = .37, *p* < .01] (Barr, 2011). Death avoidance (or avoiding thoughts of death) was significantly positively correlated with positive changes in outlook in both mothers [(*r* = .31, *p* < .05] and fathers [*r* = .44, *p* =  < .001].

With these findings, Barr (2011, 2015) suggests that parents who actively feared and avoided the idea of death were more likely to experience PTG in the aftermath of their child’s admission to intensive care. These findings reinforce previous literature that has found existential emotions to be adaptive as well as maladaptive (Cozzolino, 2006; Park et al., 2005; Tangney & Fischer, 1995).

#### Other Demonstrated Associations with PTG

Four final relationships were uncovered in this review, between PTG and (1) maternal identity, (2) marital adaptation, (3) guilt-proneness and (4) religiosity. Mothers of children with a higher sense of maternal identity in the early months of life were significantly less likely to experience positive growth [*p* < .05] (Miles et al., 1999). However, higher personal growth 1 year following the child’s admission was associated with better marital adaptation immediately following admission [*r* = .30, *p* < .05] (Taubman-Ben-Ari et al., 2010). Barr (2011) found that guilt-proneness in fathers is significantly positively associated with PTG [*r* = .25, *p* < .05]. In this case, Barr (2011) assumes this to be adaptive guilt, which has been previously associated with empathy, altruism and perspective taking (Tangney & Fischer, 1995). Finally, Brelsford et al (2020) have demonstrated associations between PTG and positive religious coping [*r* = .41, *p* < .05], spiritual disclosure [*r* = .43, *p* < .05], theistic sanctification (the perception of God’s presence in the parent–child relationships) [*r* = .52, *p* < .05] and non-theistic sanctification (the perception of the sacred in the parent–child relationship) [*r* = .73, *p* < .001].

## Discussion

The experience of one’s child being admitted to intensive care represents a significant traumatic event in the life of a parent. Whilst the high rate of resulting post-traumatic stress symptoms has been previously documented amongst this population of parents (Bronner et al., 2010; Lefkowitz et al., 2010; Rees et al., 2004), the positive change parents undergo following their child’s discharge from the intensive care unit has been a less investigated topic. The present review reveals that the phenomenon of PTG is highly prevalent amongst these parents and has strong links to parental psychological well-being and patterns of adaptive coping.

The finding that PTG is more common amongst mothers when compared to fathers is unsurprising. Previous research demonstrates that the female gender is a significant predictor of PTG in parents of critically ill children (Hungerbuehler et al., 2011). However, whether the reason for this lies within the maternal parenting role, or the willingness to be in contact with distressing thoughts, feelings and images, which Kashdan and Kane (2011) posit serves as a catalyst for the development of PTG, warrants further examination. It must also be noted that most studies in the present review examined maternal PTG. It remains important that future research in the area of PTG seeks to include a greater number of fathers.

Perhaps more interesting is the conflicting evidence regarding the possible curvilinear relationship between PTSD and PTG uncovered within the findings of this review (Colville & Cream, 2009; Rodriguez-Rey & Alonso-Tapia, 2017). One possible explanation for the divergent findings of this review lies in the significant relationship identified between PTG and parents’ perceptions of the severity of their child’s illness (Rodriguez-Rey & Alonso-Tapia, 2017, 2018). Given that the time spent in intensive care can be an acutely distressing experience that may impact on parents’ perceptions of illness severity, future research should endeavour to track both perceptions of illness severity, and PTG, over time, to assess whether this variable may moderate the relationship between PTSD and PTG. This review further highlights the need for longitudinal research with parents following their child’s discharge from intensive care to examine the trajectory of the PTG experienced.

Davydow et al.’s (2010) review of the factors associated with PTSD symptoms in parents of children admitted to intensive care highlighted that psychological variables were more strongly associated with subsequent PTS symptoms than demographic or medical variables. These psychological variables included retrospective reports of stress experienced during admission (Baluffi et al., 2004; Colville & Gracey, 2006) and parents’ perceptions of how life-threatening their child’s illness is (at the time of admission) (Baluffi et al., 2004). These findings suggest that, in terms of predicting psychological outcome, subjective experience is more important than objective aspects of the ICU experience—i.e. how something is experienced is more of a predictor of future distress than what is experienced (Colville & Pierce, 2012). Indeed, it is widely acknowledged that acute stress can result in significant distortion effects on both one’s current perception of an event and recollections in memory (Hancock & Weaver, 2005; Mather & Sutherland, 2011). Findings uncovered in the present review suggest that this same underlying process may be at play in the development of PTG, as in the development of PTSD, over time.

The strategies employed to cope with the post intensive care experience were another significant factor associated with the development of PTG. The adaptive nature of existential emotions, previously often thought to be maladaptive, has been demonstrated (Barr, 2015). In the same way that Cozzolino (2006) postulates that contemplating one’s own mortality can promote personal growth; the existential emotion of fearing death was significantly associated with PTG (Barr, 2011).

Prior to Barr’s (2015) novel finding, death avoidance was widely believed to hamper facets of personal growth (Cozzolino, 2006; Tomer et al., 2007). However, this finding suggests that parents of children previously admitted to intensive care may use avoidance of thoughts of death as an effective coping strategy. One theory why this strategy may promote PTG suggests that this is due to the experience of their child’s ICU admission being uniquely mortality salient (i.e.—resulting in an awareness that death is inevitable) (Lykins et al., 2007). The traumatic experience of their child’s admission to intensive care may cause parents to appraise the fragility of life, thus resulting in positive appraisal and PTG. Future research should seek to confirm this finding and more closely examine the processes involved.

### Strengths and Limitations of the Review

To the author’s knowledge, this is the first systematic review to synthesis evidence relating to the factors associated with PTG in a population of parents whose children have previously been admitted to intensive care. The review employed a comprehensive screening and quality appraisal process which may be viewed as a strength.

Despite this, the present review was not without limitations. Due to the heterogeneity of the variables examined within the studies, a narrative synthesis was undertaken with no meta-analytic component. Thus, the review provides a mainly descriptive account of the findings. Many studies included in this review were deemed to be of “fair” quality (*n* = 6), with some concerns regarding risk of bias. The findings of the study should be considered in the context of their assessed methodological rigour. Finally, this review only included studies employing a quantitative design. Research using qualitative methods may have contributed to our understanding of PTG in this population.

### Clinical Implications

The positive links established between PTG and the psychological well-being of parents following their child’s admission to intensive care are of clinical relevance. Future research should seek to rigorously evaluate clinical interventions that seek to promote PTG amongst these parents, as evidence from this review suggests that this may in turn increase psychological well-being.

Considering the well-documented link between parent psychopathology and child psychopathology, and the observation that almost half of all children admitted to intensive care are aged below 1 year (Paediatric Intensive Care Audit Network, 2005), it may be most appropriate to provide such an intervention at parent level. Interventions to promote PTG in parents therefore denote a valuable investigation when seeking to improve family-wide psychological well-being. Such interventions should be developed and evaluated in order to facilitate growth and positive outcomes for parents of children previously admitted to intensive care.

### Future Research

The findings of the present review highlight the heterogeneity amongst the factors examined when seeking to investigate PTG in this population of parents. Future research should seek to employ greater homogeneity when examining what predicts PTG amongst parents whose child has been admitted to intensive care. More focused research would help in the design of a feasible parent intervention to promote PTG. Additionally, fathers were underrepresented in the present review, when compared to mothers. Fathers are historically underrepresented in psychology and healthcare research (Seiffge-Krenke, 2002; Garfield & Isacco, 2012), despite their important role in child development (Sarkadi et al., 2008). Future research in the area of PTG should seek to include more paternal voices.

Finally, this area of research would benefit greatly from more studies employing a longitudinal design. Helgeson et al. (2006) have highlighted that PTG-related outcomes often take over 2 years to manifest. None of the included studies in this review have incorporated a timeframe for observing PTG greater than 16 months, with the median timeframe being 9.5 months following ICU admission. Future research should aim to fill this gap in the literature in a bid to investigate the long-term trajectory of PTG in this population of parents.

## Conclusion

The present systematic review demonstrates that PTG is a common positive outcome for parents following the exceptionally distressing event of having a child admitted to the intensive care unit. Whilst mothers more commonly experienced PTG, psychological factors were more commonly associated with PTG in comparison with demographic and clinical factors. Such psychological factors include post-traumatic stress, coping and perceived severity of their child’s illness. This suggests that parents’ subjective experience of intensive care may be greater associated with PTG than the objective reality. This is an important consideration when seeking to develop psychological interventions for parents of children admitted to intensive care, suggesting that, for example, it may be beneficial to screen parents’ levels of subjective distress whilst in ICU with their child. Future research would benefit from examining variables of greater homogeneity, and employing longer timeframes, when investigating PTG amongst parents of children previously admitted to intensive care.

## Supplementary Information

Below is the link to the electronic supplementary material.

Supplementary file1 (DOCX 13 kb)

Supplementary file2 (DOCX 19 kb)
