# Supplementary material for: A Systematic Review of the Factors Associated with Post-Traumatic Growth in Parents Following Admission of Their Child to the Intensive Care Unit
Source: J Clin Psychol Med Settings. 2022 May 8;29(3):509–37. doi: 10.1007/s10880-022-09880-x (PMC9399044; doi:10.1007/s10880-022-09880-x)
Supplement: Supplementary file 1 — Supplementary file1 (DOCX 13 kb) [file 10880_2022_9880_MOESM1_ESM.docx]

**Supplementary Table 1:** Database search terms

| Databases: PubMed, Medline, Web of Science, PsycINFO, CINAHL, PTSDpubs and EMBASE | |
| --- | --- |
| Search strategy: | |
| 1. | posttrauma* growth |
| 2. | post-trauma* growth |
| 3. | PTG |
| 4. | personal growth |
| 5. | positive growth |
| 6. | **1 or 2 or 3 or 4 or 5** |
| 7. | parent* |
| 8. | mother* |
| 9. | caregiver* |
| 10. | **7 or 8 or 9** |
| 11. | paediatric critical illness |
| 12. | pediatric critical illness |
| 13. | paediatric critical care |
| 14. | pediatric critical care |
| 15. | critically-ill child* |
| 16. | paediatric intensive care unit |
| 17. | pediatric intensive care unit |
| 18. | PICU |
| 19. | neonatal intensive care unit |
| 20. | NICU |
| 21. | **11 or 12 or 13 or 14 or 15 or 16 or 17 or 18 or 19 or 20** |
| 22. | **6 and 10 and 21** |
| Final search terms: “[posttrauma* growth OR post-trauma* growth OR PTG OR personal growth OR positive growth] AND [parent* OR mother* OR father* OR caregiver*] AND [paediatric critical illness OR pediatric critical illness OR paediatric critical care OR pediatric critical care OR  critically-ill child* OR paediatric intensive care unit OR pediatric intensive care unit OR PICU OR neonatal intensive care unit OR NICU]” | |
