# Supplementary material for: A Systematic Review of the Factors Associated with Post-Traumatic Growth in Parents Following Admission of Their Child to the Intensive Care Unit
Source: J Clin Psychol Med Settings. 2022 May 8;29(3):509–37. doi: 10.1007/s10880-022-09880-x (PMC9399044; doi:10.1007/s10880-022-09880-x)
Supplement: Supplementary file 2 — Supplementary file2 (DOCX 19 kb) [file 10880_2022_9880_MOESM2_ESM.docx]

**Supplementary Table 2:** Tools used in the assessment of psychological factors

| **Variable** | **Measure** | **Measure Author(s)** | **Number of studies** |
| --- | --- | --- | --- |
| **Parental Stress** | Perceived Stress Scale  [PSS] | Cohen, Kamarck, & Mermelstein (1983) | 2 |
|  | Parental Stressor Scale: PICU  [PSS: PICU] | Carter & Miles (1989) | 2 |
|  | Parental Stressor Scale: NICU  [PSS: NICU] | Miles, Funk, & Carlson (1993) | 1 |
|  | Parental Stressor Scale: Infant Hospitalisation [PSS: IH] | Miles, Funk, & Carlson (1993) | 1 |
|  | Stanford Acute Stress Reaction Questionnaire [SASRQ] | Cardeña, Koopman, Classen, Waelde, & Spiegel (2000) | 1 |
|  | The Family Inventory of Life Events and Changes (FILE) | McCubbin & Thompson (1987) | 1 |
| **Posttraumatic Stress** | Impact of Events Scale - Revised [IES-R] | Weiss & Marmar (1997) | 3 |
|  | Impact of Events Scale [IES] | Horowitz, Wilner, & Alvarez (1979) | 1 |
|  | Davidson Trauma Scale [DTS] | Davidson et al. (1997) | 1 |
| **Psychological Wellbeing & Mental Health** | Ryff’s Psychological Well-Being Scales [PWB] | Ryff & Keyes (1995) | 2 |
|  | Mental Health Inventory [MHI] | Veit & Ware (1983) | 2 |
| **Depression** | Hospital Anxiety and Depression Scale [HADS] | Zigmond & Snaith (1983) | 2 |
|  | Center for Epidemiologic Studies Depression Scale [CES-D] | Radloff (1977) | 2 |
|  | Depression Anxiety Stress Scale [DASS] | Lovibond & Lovibond (1995) | 1 |
| **Coping** | COPE Inventory | Carver, Scheier, & Weintraub (1989) | 2 |
|  | Coping Inventory for Stressful Situations [CISS] | Endler & Parker (1999) | 1 |
|  | Revised Ways of Coping Questionnaire [WOCQ] | Folkman, Lazarus, Dunkel-Schetter, DeLongis, & Gruen (1986) | 1 |
|  | The Brief Religious Coping Scale [RCOPE] | Pargament, Feuille, & Burdzy (2011) | 1 |
| **Attitudes towards Death** | Death Attitude Profile – Revised [DAP-R] | Wong, Reker, & Gesser (1994) | 1 |
|  | Collett-Lester Fear of Death Scale [CLFDS] | Lester (1990) | 1 |
| **Social and Emotional Support** | Multidimensional Scale of Perceived Social Support [MSPSS] | Zimet, Dahlem, Zimet, & Farley (1988) | 1 |
|  | Support Functions Scale [SFS] | Dunst, Trivette, & Deal (1988) | 2 |
| **Anxiety** | Hospital Anxiety and Depression Scale [HADS] | Zigmond & Snaith (1983) | 2 |
| **Worry about Child** | Child Health Worry Scale [CHWS] | Miles (1998) | 1 |
|  | Mothers’ Feelings towards their Baby Questionnaire | Levy-Shiff, Sharir, & Mogilner (1989) | 1 |
| **Attachment Style** | Experiences in Close Relationships Scale [ECRS] | Brennan, Clark, & Shaver (1998) | 2 |
| **Guilt & shame-proneness** | Test of Self-Conscious Affect-3 (TOSCA) | Tangney & Dearing (2002) | 1 |
| **Sense of Control** | Sense of Mastery Scale [SOM] | Pearlin, Lieberman, Menaghan, & Mullan (1981) | 1 |
| **Satisfaction with Family Life** | Family Apgar Scale | Smilkstein, Ashworth, & Montano (1982) | 1 |
| **Maternal Identity** | Maternal Identity Scale: Critically Ill Infant [MIS] | Miles (1998) | 1 |
| **Maternal Presence** | Behavioural observations of mother-infant interactions | N/A | 1 |
| **Maternal Competence** | Behavioural observations of mother-infant interactions *plus* the Home Observation for Measurement of the Environment [HOME] | *of the HOME Inventory:*  Caldwell & Bradley (1984) | 1 |
| **Resilience** | Brief Resilience Scale [BRS] | Smith et al. (2008) | 1 |
| **Emotions during Admission** | Modified Differential Emotions Scale [mDES] | Fredrickson, Tugade, Waugh, & Larkin (2003) | 1 |
| **Self-esteem** | Self-esteem Scale [SES] | Rosenberg (1979) | 1 |
| **Marital adaptation** | Evaluating and Nurturing Relationship Issues Communication and Happiness Scale [ENRICH] | Fowers & Olson (1989) | 1 |
| **Infant Temperament** | Infant Characteristics Questionnaire [ICQ] | Bates, Freeland, & Lounsbury (1979) | 1 |
| **Religiosity** | Spiritual Disclosure Scale [SDS] | Brelsford & Mahoney (2008) | 1 |
|  | Manifestation of God in Parent–Child Relationships scale | Mahoney et al. (1999) | 1 |
|  | Sacred Qualities Scale | Mahoney et al. (1999) | 1 |
